# Medical and Dental Libraries

**Published:** 1903-08

**Authors:** Alice M. Steeves

**Affiliations:** Boston, Mass.


### MEDICAL AND DENTAL LIBRARIES.[1]

BY ALICE M. STEEVES, D.D.S., BOSTON, MASS.

In this age of strenuous activity and rapid advancement in the scientific and literary world the need of systematic library work has in a measure been anticipated.

As early as 1760 Benjamin Franklin saw the need of general public libraries, and since that time libraries have been founded in nearly all of our cities and towns.

The mother profession (Medicine) has been more than successful in its work in this direction, and during the past twenty years, under the generalship of Billings, Chadwick, Dewey, Spivak, and others, medical libraries have been organized in connection with medical societies, library associations have been formed, and splendid library buildings built and equipped. Medical departments have been organized in public libraries, and even State and local boards of health have their library departments.

On receiving an invitation from your genial secretary to contribute to your programme, my first thought was, What have I to

---

[1] Abstract of a paper read before the American Medical Association, Section on Stomatology, New Orleans, May 5 to 8, 1903.

write about? And after thinking over some of my experiences of the past year, it seemed to me that this story might be of some interest to many.

A year ago I came to Boston to take the prescribed examination of the examining board for dental registration, and feeling that I ought to do a little preparatory reading (it being about five years since my graduation), I looked around for the proper literature.

I knew of Boston's magnificent Public Library and of her splendid Medical Library. I hastened thither, feeling sure that I should find all the books that I needed, but although a nucleus had been formed and many files of periodicals were complete, yet the works of our modern writers on dental subjects were conspicuous by their absence.

However, I passed my examination, and resolved to try and add my mite of energy to the much-needed work of arousing an interest in dental literature.

In my preliminary work on this subject I have interviewed the librarian of the Boston Medical Library, Dr. J. R. Chadwick, and some of his colleagues, together with Dr. Jacob Williams and other librarians of the various dental societies to whom the library is indebted for the dental literature it now possesses, and all are enthusiastic over the prospect of a well-organized dental section in the Boston Medical Library.

I also wrote to seventy-five dental colleges, asking for any information which they might be able to give. Twenty-five responded; five of this number reported a library of over fifteen hundred volumes of books and periodicals, with current dental periodicals for the reading-room. Sixteen reported libraries containing from one hundred to eight hundred books. Four of the five reported catalogued libraries, with the Dewey card system, and reading-rooms for the students.

The need of more interest in the work, lack of funds, and inadequate space was the complaint of all, except Ann Arbor and the Northwestern University Dental School.

Dr. Taft reports from Ann Arbor:

"Our library was started twenty-five years ago. It contains nearly all the text-books that have been written in the English language upon the subject of dentistry. It also contains the translations of many books in other languages, and also some that have not been translated.

"In addition we have nearly complete files of all the dental journals that have been published in the country, and also the files of the transactions of those societies which have published the minutes of their meetings. There aıe about fifteen hundred bound volumes and five thousand pamphlets.

"We regard our library as a very valuable adjunct to the work of teaching; the regents give us five hundred dollars a year for keeping up and extending our library (we would hardly know how to keep school without our library)."

The Northwestern University reports that its library includes about two thousand nine hundred volumes, at a cost of about nine thousand dollars; this of course includes furnishings of a reading-room. The library was started in 1898 by Dr. Theodore Menges. It is now under the management of Dr. G. V. Black, and is strictly a dental library, the purpose being to include dental literature, with books on such allied sciences as pertain to the dental art. "We find it indispensable to our students and one of the most valuable additions to our general equipment."

Only two reported that the Medical Library was supplied with dental literature and that the medical and dental students used the same reading-room.

The advantages to the dental specialist of a general medical library are many; in this age of specialties, where the line of duty has been so closely drawn, it is many times necessary for us to confer with the rhinologist, ophthalmologist, neurologist, gastrologist, or perhaps with the general practitioner, in regard to our patients.

The neurasthenic may be unable to endure the fatigue and fear of the slightest dental operation, but the teeth ought to be saved.

What shall we do, fail in our duty to the patient and acknowledge ourselves not worthy the place we occupy in the scientific world, or shall we grind, grind? Keep abreast of the times and be able to co-operate with any department of medicine.

However, it is not always easy to convince some of our Æsculapian friends that they do sometimes need our co-operation, but year by year we find this feeling passing away, and we are now welcomed in their ranks if we will only meet them half-way.

To the dental student the library is equally important for reference and study, and that the student may gain the most benefit from his work a good reading-room is necessary and access to the books during any part of the day that he may be free to study.

However, it seems that it would be well when practical to work with an already existing medical library.

I cannot do better now than to give you a revised edition of some important suggestions of Dr. George M. Gould, of Philadelphia:

1. The systematization and practical carrying out of a method to effect the exchange of duplicate books whereby such volumes not needed by one library may by exchange or purchase find their way to the library heretofore without them.

2. A committee to make effective some means of bringing to public medical libraries the books of dentists deceased or retiring from practice.

3. A list of all the dental societies in the world which publish reports or transactions, and to solicit gifts or exchange of these reports to each one of our library sections.

4. To endeavor to bring the attention of wealthy individuals and of older physicians and dentists to the present sad state of dental libraries, and the tremendous possibilities of good that would come from endowing, founding, and organizing for the purpose of bringing medical (I mean dental, too) literature within the practical use of the profession.

At present rich men, and women, too, seem to be devoting their millions to purposes other than scientific medicine. During the past year a step has been taken in the right direction, and Harvard Medical College has received a munificent gift; but, sad to relate, adequate provision has not yet been made for the dental department. However, Harvard will be true to the first essentials of life—thorough mastication, perfect digestion, perfect assimilation, hence perfect scholars.

Let us show them what good they could do by bringing to every individual dental specialist trying to save the health and comfort of his patients the experiences of the world's workers.

